# Antihypertensive drugs and arteriovenous fistula flow rates among patients on maintenance hemodialysis in Egypt

**DOI:** 10.1080/0886022X.2026.2670067

**Published:** 2026-05-31

**Authors:** Enass Elsayed, Khaled M. Kotb, Mohamed Abdel-Salam El-Awady, Manar Samy M., Omnia Ali, Mohammed Abdel Gawad

**Affiliations:** a Department of Nephrology, Ahmad Maher Teaching Hospital, GOTHI, Cairo, Egypt; b Brigham and Women’s Hospital, Boston, MA, USA; c Harvard Medical School, Boston, MA, USA; d Clinical Pharmacy and Pharmacy Practice Department, Faculty of Pharmacy, Mansoura University, Mansoura, Egypt; e Department of Environmental and Occupational Health, School of Public Health, Texas A&M University, College Station, TX, USA; f Specialized Airforce Hospital, Cairo, Egypt; g Artificial Kidney Department, El-Gamaliya Hospital, Dakahlia, Egypt; h Clinical and Chemical Pathology Department, Ahmed Maher Teaching Hospital, GOTHI, Cairo, Egypt; i Nephrology Unit, Internal Medicine Department, School of Medicine, Newgiza University (NGU), Giza, Egypt

**Keywords:** Hemodialysis, arterio-venous fistula, antihypertensive drugs, vasodilators, flow rate

## Abstract

Vascular access dysfunction is a major cause of morbidity among maintenance hemodialysis (HD) patients. Calcium channel blockers (CCBs) may affect vascular remodeling; however, their impact on arteriovenous fistula (AVF) flow rate remains uncertain. We evaluated the association between antihypertensive therapy, particularly CCBs, and AVF flow rate in maintenance HD patients. In this cross-sectional study, 79 HD patients were categorized into those receiving antihypertensive medications (*n* = 58) and those not (*n* = 21) – subgroup analyses compared CCBs (*n* = 26) and beta blocker (BBs) users (*n* = 23). AVF Doppler ultrasound parameters, including flow rate, were analyzed. Multivariable regression assessed associations between treatment groups and AVF flowrate adjusting for age, sex, dialysis vintage, diabetes mellitus (DM), calcium level, and cardiac disease. Median AVF flow rate was comparable between CCBs users and BBs users (971 [803–1739] vs. 1227 [937–1519] mL/min, *p* = 0.348). In adjusted analyses, CCBs therapy was not independently associated with AVF flow rate (β = −8.41 mL/min; 95% CI: −432.07,415.25; *p* = 0.968) or other vascular parameters. Age was inversely associated with AVF flow (β = −14.2; *p* = 0.013), while DM showed a non-significant trend toward higher flow (β = 306; *p* = 0.17). Older age was inversely associated with prior AVF failure (OR = 0.95; *p* = 0.024), whereas vascular access type and duration were not significant. CCBs use was not associated with differences in AVF flow rate, however, its use may confer protection against AVF failure. Prospective studies are needed to confirm these findings and explore effects of antihypertensive classes on vascular access outcomes.

## Introduction

While arteriovenous fistulas (AVFs) are often preferred for maintenance HD due to their potential for high long-term patency and lower complication rates, current guidelines emphasize an individualized ‘ESRD Life Plan’. This approach prioritizes the ‘right access for the right patient’ based on clinical circumstances and patient goals, rather than a universal ‘fistula first’ mandate [1]. Successful AVF function depends not only on surgical technique [2,3] and vessel quality [4–6] but also on hemodynamic [7,8] and systemic factors. These systemic elements include age, underlying comorbidities such as diabetes and cardiac insufficiency, and biochemical markers like albumin and lipid levels that influence flow, remodeling, and maturation [5,9]. Hypertension is highly prevalent among end-stage renal disease (ESRD) patients, affecting over 80% of those on dialysis [10]. Antihypertensive medications, particularly beta-blockers (BBs) and calcium channel blockers (CCBs), are widely used in this population to control blood pressure and reduce cardiovascular risk [11]. However, these agents may also influence vascular tone [12,13], endothelial function [14,15], and intravascular volume [13,16], thereby potentially impacting AVF hemodynamics [17] and complication risk [13,15,18].

While BBs reduce cardiac output and sympathetic tone, CCBs exert vasodilatory effects on peripheral arteries and are postulated to improve AVF patency by enhancing arterial inflow [11]. However, data on the comparative impact of specific antihypertensive classes on AVF flow dynamics and structural complications remain limited and inconclusive. Some studies suggest beneficial effects of CCBs on AVF maturation [19], while others have reported no significant association between antihypertensive use and vascular access outcomes [20,21].

In addition, the interaction between antihypertensive therapy and AVF-related complications such as thrombosis, aneurysmal dilation, or outflow obstruction remains underexplored. Clarifying these associations is essential for optimizing individualized antihypertensive management in dialysis patients, particularly given the delicate balance between maintaining adequate blood pressure control and ensuring sufficient AVF perfusion.

Therefore, this study aimed to evaluate the association between antihypertensive therapy – particularly BBs and CCBs – and AVF flow parameters and complications in HD patients.

## Methods

### Study design and population

**Figure 1.**
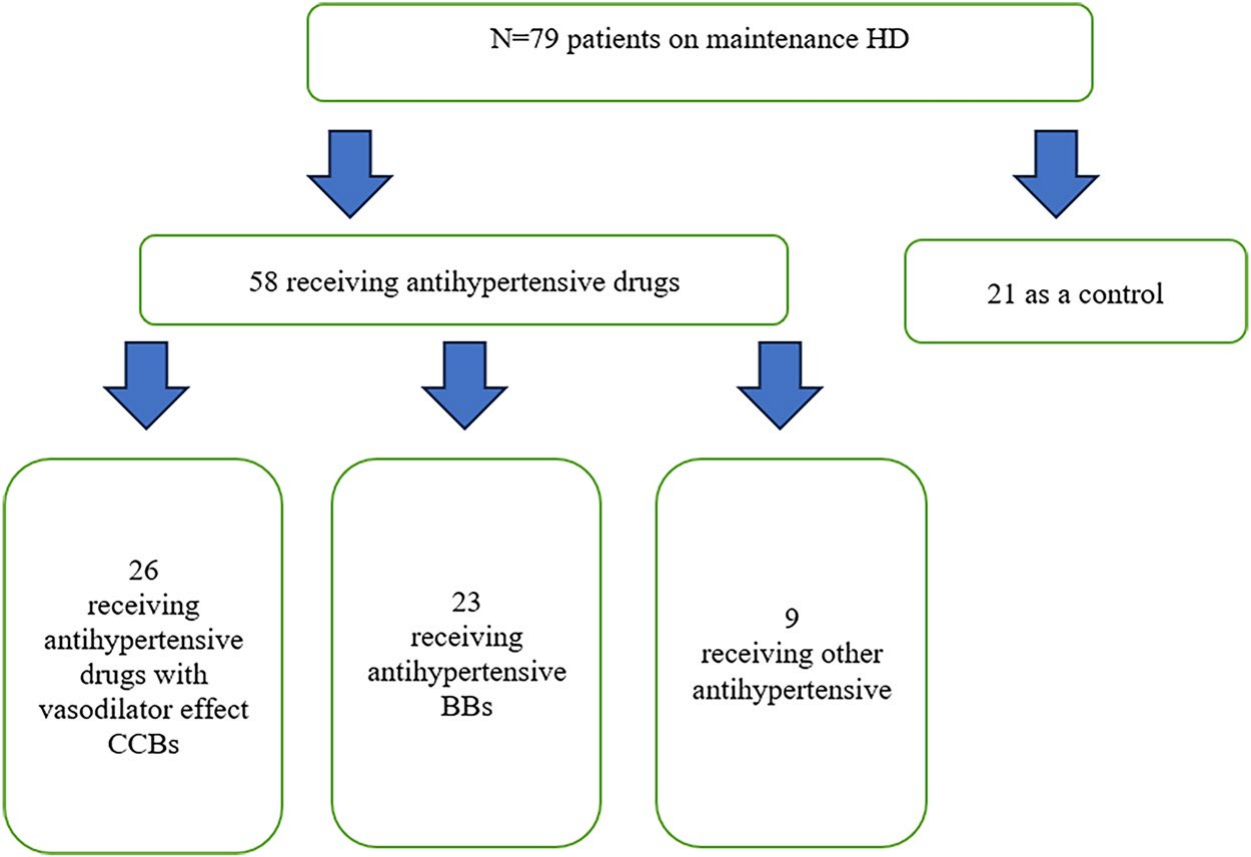
Flow chart of study participants.

This hospital-based cross-sectional study was conducted among 79 Egyptian patients on maintenance HD in Ahmad Maher Teaching Hospital, Cairo, Egypt, with 58 receiving antihypertensive drugs, and 21 not receiving antihypertensive drugs (flowchart represented in Figure 1), to evaluate the association of antihypertensive therapy and arteriovenous fistula flow rate.

Hypertension was defined according to the KDIGO 2021 guidelines as a persistent pre-dialytic blood pressure of >140/90 mmHg. Participants in the control group were those who maintained blood pressure below this threshold without pharmacological intervention and had no history of prescribed antihypertensive therapy for at least six months prior to the study [22].

The study included adults older than 18 years old with end-stage kidney disease (ESKD) on regular HD using arteriovenous fistula, receiving antihypertensive medications to control hypertension, and non-hypertensive patients on maintenance HD as a control group. Patients unwilling to participate, aged < 18 years old, patients with AKI, earlier stages of chronic kidney disease CKD, patients without an arteriovenous fistula were excluded from the study Furthermore, patients in the control group were excluded if they were receiving other vasoactive medications, such as nitrates or alpha-blockers for non-hypertensive indications (e.g., ischemic heart disease or benign prostatic hyperplasia), to ensure no confounding pharmacological effects on AVF flow rate. Antihypertensive medication use was initially collected *via* patient interviews. To minimize recall bias, these self-reported data were cross-verified with the patients’ electronic medical records and the physician-signed medication sheets maintained at the dialysis unit. Only medications taken consistently for at least six months prior to the AVF flow measurement were included in the analysis. Vascular access assessment was performed using Color Doppler Ultrasound (GE Logiq E series). Flow volume (FV) was measured in a straight, non-branching segment of the draining vein of the AVF. The cross-sectional area and time-averaged velocity were used to calculate flow, ensuring measurements were taken away from areas of turbulence. All examinations were performed by a single experienced radiologist to minimize inter-observer variability. The data received, compiled, cleaned, coded, and subsequently analyzed.

### Sample size

The sample size was calculated using Stata (version 17.0). Assuming a mean arteriovenous fistula (AVF) flow rate of 1000 mL/min in the control group and an expected increase to 1300 mL/min among patients receiving CCBs, with a standard deviation (SD) of 400 mL/min, a two-sided α of 0.05, and 80% power, the minimum required sample size was 29 participants per group (total = 58) to detect a 300 mL/min difference in mean AVF flow rates between the two groups [23,24]. We focused on CCBs use as the primary exposure for power analysis because CCBs have a distinct pharmacological mechanism (calcium channel blockade in vascular smooth muscle) that we hypothesized would have the most significant impact on AVF flow rates compared to other drug classes.

### Exposure variables

Our exposure was using any antihypertensive medications, including CCBs, BBs, renin-angiotensin system (RAS) inhibitors (including angiotensin-converting enzyme inhibitors [ACEIs] and angiotensin II receptor blockers [ARBs]), alpha-blockers, or centrally acting drugs, with CCBs as our primary exposure of interest.

### Outcome variables

The primary outcome was AVF flow rate (ml/min). The secondary outcomes were AVF Diameter (cm) measured at the same straight segment of the draining vein used for flow volume calculations (approximately 2 cm from the anastomosis), AVF Depth (mm), AVF peak systolic velocity (PSV) (cm/sec), Draining Vein PSV (cm/sec), Draining Vein Diameter (mm), Outflow Obstruction, and Aneurysmal Dilation.

### Statistical analysis

We examined data graphically; normally distributed data were represented as mean (± standard deviation), while median (Interquartile range) was used to represent non-normally distributed variables. Frequencies and corresponding proportions were used to represent categorical variables. Continuous variables were compared using the independent t-test for normally distributed data and the Mann-Whitney U test for non-normally distributed variables. Categorical variables were analyzed using the chi-square test or Fisher’s exact test, as appropriate. The Shapiro-Wilk test was used to assess the normality of data distribution.

Multivariable linear regression was used to assess the association between antihypertensive drugs and AVF flow rates. Additionally, univariate and multivariable linear regression analyses were conducted to identify predictors associated with AVF flow rates among patients on HD using three distinctive models: a demographic model (Model 1), including age, sex, smoking, and dialysis duration; a comorbidity model (Model 2), including diabetes mellitus (DM), cardiac disease, and calcium abnormality; and a vascular access-related model (Model 3), which included AVF vintage (years since creation), access type (radio-cephalic vs. brachio-cephalic), and access location (upper arm vs. forearm).

Variables were chosen based on a combination of clinical significance as identified in previous literature (factors known to influence vascular remodeling and flow rate, e.g., age, diabetes mellitus, and dialysis vintage) and statistical criteria (variables with a p-value < 0.20 in univariate analysis). This structured approach ensures that the most relevant potential confounders were accounted for in our models.

Statistical significance was set at a two-tailed P-value <0.05. All analyses were conducted using the Stata 17 statistical package (StataCorp LLC, College Station, TX).

## Results

### Baseline characteristics

A total of 79 patients on maintenance HD were included, comprising 58 (73.4%) receiving antihypertensive medications and 21 (26.6%) without antihypertensive therapy (control group) (Figure 1). Hypertension was the predominant underlying etiology of ESKD (59.49%, *n* = 47), followed by unknown causes (10.13%, *n* = 8) and DM (8.86%, *n* = 7). Other etiologies included analgesic/cortical necrosis (6.33%, *n* = 5), Autosomal Dominant Polycystic Kidney Disease (ADPKD) (5.06%, *n* = 4), pre-eclampsia (3.80%, *n* = 3), and glomerulonephritis, Systemic Lupus Erythematosus (SLE), and tubulointerstitial disease (2.53% each, *n* = 2) (Supplementary Figure 1). Patients in the antihypertensive group were comparable to the control group regarding age and gender, although numerically slightly older (mean age 52.1 ± 13.6 years vs. 49.8 ± 13.1 years, *p =* 0.529) and more often male (62.1% vs. 47.6%, *p =* 0.250). The prevalence of hepatitis C (14.6% vs. 15.0%, *p = 0.961*), smoking (13.8% vs. 6.3%, *p =* 0.414), and diabetes mellitus (17.2% vs. 9.5%, *p =* 0.399) did not differ significantly between groups. However, cardiac disease was more frequent among those on antihypertensive therapy (17.2% vs. 0%, *p =* 0.042). The mean duration of dialysis was similar in both groups (7.3 ± 5.2 years vs. 7.8 ± 4.8 years, *p =* 0.761). The median AVF vintage was longer in the antihypertensive group (5 [2–7] years vs. 2 [1.5–10] years, *p* = 0.418), though not statistically significant. There were no significant differences in AVF site, type, or flow parameters, with mean flow rates of 1309 ± 650 mL/min in the antihypertensive group versus 1387 ± 622 mL/min in controls (*p =* 0.411).

Blood pressure levels were comparable, with mean systolic and diastolic values of 147.1 ± 19.9 mmHg and 80.6 ± 10.8 mmHg, respectively, in the antihypertensive group (*p =* 0.122 and *p =* 0.409). Among hematological parameters, MCV was modestly lower in the antihypertensive group (78.70 vs. 83.79 fL, *p* = 0.022), which may reflect differences in underlying nutritional status—particularly micronutrient deficiencies such as iron, vitamin B12, or folate—commonly observed in ESRD patients on maintenance hemodialysis. Correspondingly, ferritin levels were significantly higher in the control group compared to the antihypertensive group (865.08 ± 533.27 vs. 414.96 ± 451.74 ng/mL; *p* = 0.007), which may reflect differences in iron supplementation practices or inflammatory status between groups. Neither of these differences was considered clinically relevant to AVF hemodynamics, and neither was adjusted for in subsequent regression models (Table 1).

**Table 1.** Baseline characteristics by antihypertensive use.

Characteristic	Level		Total	N	No Antihypertensive (*N* = 21)	N	Antihypertensive (*N* = 58)	P-value
**Demographics**								
Age, mean (SD)		73	51.58 (13.42)	17	49.76 (13.12)	56	52.13 (13.58)	0.529
Hepatitis C, n (%)	Yes	75	11 (14.67%)	20	3 (15.00%)	55	8 (14.55%)	0.961
Sex, n (%)	Male	79	46 (58.23%)	21	10 (47.62%)	58	36 (62.07%)	0.250
Smoking, n (%)	Yes	74	9 (12.16%)	16	1 (6.25%)	58	8 (13.79%)	0.414
Dialysis Duration (years), mean (SD)		71	7.41 (5.12)	14	7.79 (4.82)	57	7.32 (5.23)	0.761
Dialysis Duration categories, n (%)	<2 years	79	5 (6.33%)	21	1 (4.76%)	58	4 (6.90%)	0.585
	2–5 years	73	25 (31.65%)		5 (23.81%)		20 (34.48%)	
	>5 years	75	49 (62.03%)		15 (71.43%)		34 (58.62%)	
**Comorbidities**								
DM, n (%)	Yes	79	12 (15.19%)	21	2 (9.52%)		10 (17.24%)	0.399
Hypertension, n (%)	Yes		64 (81.01%)		8 (38.10%)	58	56 (96.55%)	**<0.001** *
Cardiac Disease, n (%)	Yes		10 (12.66%)		0 (0.00%)	58	10 (17.24%)	**0.042** *
**Blood Pressure**								
Systolic BP (mmHg), mean (SD)		72	144.98 (18.89)	14	138.14 (13.51)	45	147.11 (19.93)	0.122
Diastolic BP (mmHg), mean (SD)		71	79.98 (10.92)	14	77.86 (11.60)	45	80.64 (10.75)	0.409
**AVF Characteristics**								
AVF vintage, median (IQR)		43	5 (2–7)	7	2 (1.5–10)	36	5 (2–7)	0.418
AVF vintage category, n (%)	<2 years		8 (18.6%)		2 (28.57%)		6 (16.67%)	0.663
	2–5 years		17 (39.53%)		3 (42.86%)		14 (38.89%)	
	>5 years		18 (41.86%)		2 (28.57%)		16 (44.44%)	
AVF Site Laterality, n (%)	Left	67	51 (76.12%)	15	11 (73.33%)		40 (76.92%)	0.774
AVF Type, n (%)	Brachiocephalic	66	48 (72.73%)	18	14 (77.78%)	48	34 (70.83%)	0.573
	Radiocephalic		18 (27.27%)		4 (22.22%)		14 (29.17%)	
Previous Failed AVF, n (%)	Yes	70	19 (27.14%)	15	6 (40.00%)	55	13 (23.64%)	0.206
Aneurysmal Dilation, n (%)	Yes	69	22 (31.88%)	14	9 (64.29%)	55	38 (69.09%)	0.731
Outflow Obstruction, n (%)	Yes	23	22 (8.70%)	8	1 (12.50%)	15	1 (6.67%)	0.636
Edema, n (%)	Yes	53	4 (7.55%)	12	3 (25.00%)	41	1 (2.44%)	**0.009** *
**AVF Flow Parameters**								
AVF Diameter (cm), mean (SD)		4	1.6 (1.29)	2	1.00 (0.42)	2	2.20 (1.84)	0.667
AVF Depth (mm), mean (SD)		5	1.98 (.43)	2	2.20 (0.28)	3	1.83 (0.50)	0.400
AVF PSV (cm/sec), mean (SD)		48	232.82 (116.31)	11	238.63 (106.00)	37	231.09 (120.53)	0.699
Draining Vein Diameter (mm), mean (SD)		6	3.25 (3.43)	1	1.40 (-)	5	3.62 (3.70)	–
Draining Vein PSV (cm/sec), mean (SD)		72	144.04 (115.41)	19	153.74 (157.86)	53	140.56 (97.54)	0.579
Flow rate (ml/min), mean (SD)		71	1329.94 (638.88)	19	1387.17 (621.96)	52	1309.03 (649.64)	0.411
**AVF Complications**								
Complications Category, n (%)	Normal/No Abnormalities	79	45 (56.96%)	21	13 (61.90%)	58	32 (55.17%)	0.394
	Cephalic Obstruction		21 (26.58%)		3 (14.29%)		3 (5.17%)	
	Partial Thrombosis		7 (8.86%)		1 (4.76%)		7 (12.07%)	
	Calcification		4 (5.06%)		0 (0.00%)		4 (6.90%)	
	Wall Thickening		2 (2.53%)		4 (19.05%)		12 (20.69%)	
**Hematology**								
Hemoglobin (g/dL), mean (SD)		74	9.11 (1.61)	19	8.68 (1.91)	55	9.25 (1.49)	0.188
White Blood Cells (10³/µL), mean (SD)			6.76 (2.32)	19	6.22 (2.65)	55	6.94 (2.19)	0.244
Red Blood Cells (10⁶/µL), mean (SD)		71	3.55 (.62)	18	3.33 (0.54)	53	3.63 (0.63)	0.080
Hematocrit (%), mean (SD)		74	28.14 (5.34)	19	27.88 (3.65)	55	28.23 (5.84)	0.809
Mean Corpuscular Hemoglobin (pg), mean (SD)		71	27.02 (7.69)	18	30.50 (14.14)	53	25.84 (2.95)	0.051
Mean Corpuscular Hemoglobin Concentration (g/dL), mean (SD)			32.65 (1.34)	18	32.42 (0.93)	53	32.72 (1.45)	0.406
Lymphocytes (10³/µL), mean (SD)			4.73 (7.53)	18	4.24 (7.56)	53	4.90 (7.59)	0.749
Platelets (10³/µL), mean (SD)		72	274.15 (290.3)	18	382.72 (557.87)	54	237.96 (87.93)	0.331
Mean Corpuscular Volume (fL), mean (SD)		71	79.99 (8.33)	18	83.79 (8.51)	53	78.70 (7.94)	**0.022** *
Mean Platelet Volume (fL), mean (SD)		70	12.73 (15.76)	18	13.53 (17.08)	52	12.46 (15.45)	0.486
**Renal Function**								
Blood Urea Nitrogen (mg/dL), mean (SD)		72	139.66 (33.63)	18	127.78 (29.21)	54	143.63 (34.31)	0.083
Urea (mg/dL), mean (SD)		69	57.29 (27.6)	17	56.21 (29.37)	52	57.64 (27.29)	0.854
Creatinine (mg/dL), mean (SD)		74	9.48 (2.53)	19	8.91 (2.43)	55	9.68 (2.55)	0.260
**Electrolytes and Minerals**								
Calcium (mg/dL), mean (SD)		74	8.68 (.97)	19	8.69 (0.77)	55	8.68 (1.04)	0.953
Phosphorus (mg/dL), mean (SD)		70	13.97 (52.62)	19	4.48 (1.78)	51	17.51 (61.43)	0.192
Alkaline Phosphatase (U/L), mean (SD)		56	183.44 (146.11)	15	163.93 (165.51)	41	190.58 (139.90)	0.226
Albumin (g/dL), mean (SD)		72	4.96 (9.57)	18	3.74 (0.47)	54	5.37 (11.05)	0.497
**Iron Studies**								
Serum Iron (µg/dL), mean (SD)		42	85.90 (187.9)	13	71.92 (44.49)	29	92.17 (225.21)	0.096
Ferritin (ng/mL), mean (SD)			543.57 (512.83)	12	865.08 (533.27)	30	414.96 (451.74)	**0.007** *
Total Iron-Binding Capacity (µg/dL), mean (SD)		41	226.29 (49.69)	12	219.92 (63.93)	29	228.93 (43.56)	0.603

* Significant p-value.

**Figure 2.**
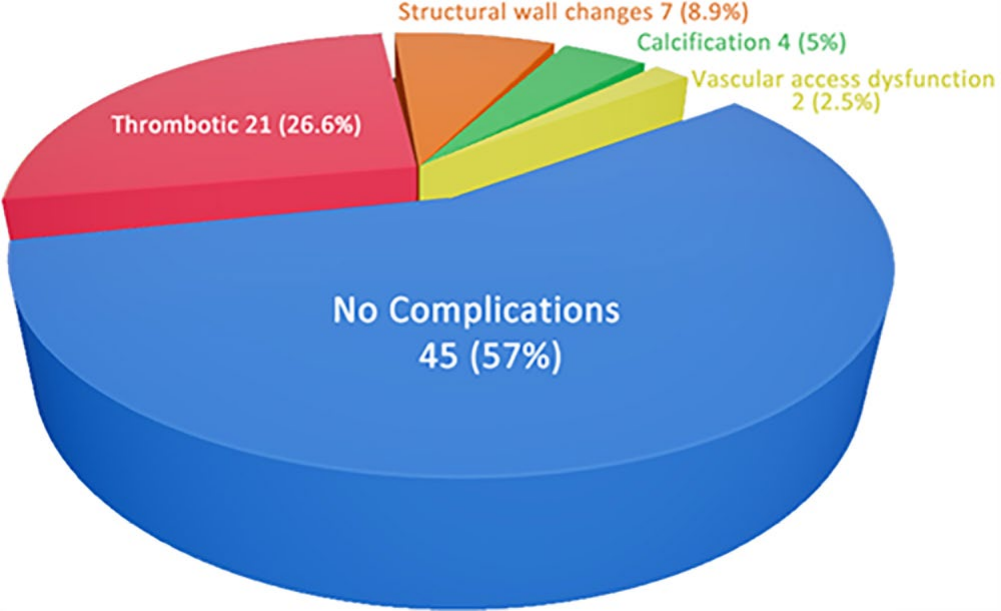
Distribution of ultrasound findings of AVF complications in study patients.

Figure 2 shows the Color Doppler ultrasound evaluation of complications, where the majority of patients showed no detectable complications, accounting for 45 cases (57%). Among the identified complications, thrombotic changes were the most frequent finding, observed in 21 patients (26.6%). Structural wall abnormalities were detected in 7 patients (8.9%), while calcification was noted in 4 patients (5%). Vascular access dysfunction without overt structural or thrombotic changes was the least common finding, occurring in 2 patients (2.5%).

### Subgroup analysis

#### Comparing the CCBs and the BBs group

Among the 58 patients receiving antihypertensive therapy, 26 (44.8%) were treated with CCBs and were compared with 23 (39.7%) patients receiving BBs. (Supplementary Figure 2 illustrates the distribution of antihypertensive medications within the study population).

CCBs-treated patients had a similar mean age as the BBs group (51.0 ± 14.4 vs. 52.5 ± 12.3 years, *p =* 0.711) and a comparable proportion of males (53.8% vs. 73.9%, *p =* 0.146). Hepatitis C infection was significantly less frequent among CCBs users (3.8% vs. 23.8%, *p =* 0.041). The CCBs group had a longer mean dialysis duration (9.4 ± 5.5 vs. 5.3 ± 3.6 years, *p =* 0.004), while the prevalence of diabetes, cardiac disease, and smoking was comparable between groups.

Systolic blood pressure was significantly lower in the CCBs group compared with the BBs group (138.6 ± 18.8 vs. 154.8 ± 18.0 mmHg, *p =* 0.009), whereas diastolic pressure showed no significant difference (*p =* 0.097). Regarding vascular access characteristics, the median AVF flow rate was 971 (803–1739) mL/min in the CCBs group compared with 1226.5 (937–1519) mL/min in the BBs group (*p* = 0.348), indicating no statistically significant difference. AVF vintage and access type were comparable between groups (*p* > 0.05). CCBs users had a higher frequency of previous failed AVFs (33.3% vs. 8.7%, *p* = 0.039), while AVF site, type, and flow parameters were comparable between groups. (Table 2).

**Table 2.** Characteristics of patients by treatment group (CCBs vs. BBs).

Variable	Level	N	CCBs (*N* = 26)	N	BBs (*N* = 23)	P-value
**Demographics**						
Age, years, mean (SD)		26	51.0 (14.4)	23	52.5 (12.3)	0.711
Sex, n (%)	Male		14 (53.8%)		17 (73.9%)	0.146
Hepatitis C, n (%)	Yes		1 (3.8%)	21	5 (23.8%)	**0.041** *
Smoking, n (%)	Yes		4 (15.4%)	23	3 (13.0%)	0.815
Dialysis duration, years, mean (SD)			9.4 (5.5)		5.3 (3.6)	**0.004** *
Dialysis Duration categories, n (%)	<2 years		0 (0.00%)		3 (13.04%)	0.074
	2–5 years		7 (26.92%)		9 (39.13%)	
	>5 years		19 (73.08%)		11 (47.83%)	
**Comorbidities**						
DM, n (%)	Yes	26	5 (19.2%)	23	3 (13.0%)	0.559
Hypertension, n (%)	Yes		25 (96.2%)		23 (100%)	0.342
Cardiac Disease, n (%)	Yes		4 (15.4%)		4 (17.4%)	0.850
**Blood Pressure**						
Systolic BP, mmHg, mean (SD)		26	138.6 (18.8)	23	154.8 (18.0)	**0.009** *
Diastolic BP, mmHg, mean (SD)			82.6 (11.4)		76.7 (9.7)	0.097
**AVF Characteristics**						
AVF Site, n (%)	Right	24	3 (12.5%)	19	6 (31.6%)	0.127
	Left		21 (87.5%)		13 (68.4%)	
AVF Type, n (%)	Brachiocephalic	19	11 (57.9%)	21	17 (81.0%)	0.112
	Radiocephalic		8 (42.1%)		4 (19.0%)	
Other Failed AVF, n (%)		24	8 (33.3%)	23	2 (8.7%)	**0.039** *
Aneurysmal Dilation, n (%)			19 (79.2%)	22	14 (63.6%)	0.243
Outflow Obstruction, n (%)		14	1 (7.1%)	–		–
AVF Diameter, cm, median (IQR)		2	2.2 (0.9–3.5)	–	–	–
AVF Depth, mm, median (IQR)		12	1.6 (1.3–1.9)	2	2.3 (2.3–2.3)	0.667
AVF PSV, cm/sec, median (IQR)			223 (122–310.3)		196 (140–270)	0.650
Draining Vein Diameter, cm, median (IQR)			5.3 (0.9–9.6)		3.3 (1.8–4.8)	1.000
Draining Vein PSV, cm/sec, median (IQR)			144 (58.5–203)		101.5 (78–179.5)	0.657
Flow, mL/min, median (IQR)			971 (803–1739)		1226.5 (937–1519)	0.348
**Complications**						
Complications Category, n (%)	Normal/No Abnormalities	26	15 (57.69%)	23	14 (60.87%)	0.301
	Cephalic Obstruction		1 (3.85%)		1 (4.35%)	
	Partial Thrombosis		3 (11.54%)		2 (8.70%)	
	Calcification		3 (11.54%)		0 (0.00%)	
	Wall Thickening		4 (15.38%)		6 (26.09%)	
**Hematology**						
Hemoglobin (HGB), g/dL, mean (SD)		26	9.2 (1.4)	23	9.4 (1.6)	0.653
White Blood Cells (WBC), x10³/µL, mean (SD)			6.8 (2.6)		7.1 (1.7)	0.569
Red Blood Cells (RBC), x10⁶/µL, mean (SD)			3.6 (0.6)		3.7 (0.7)	0.601
Hematocrit (HCT), %, mean (SD)			28.5 (4.7)		27.8 (7.5)	0.696
Mean Corpuscular Hemoglobin (MCH), pg, median (IQR)			26 (25.4–28.3)		25.3 (24.1–27.5)	0.111
Mean Corpuscular Hemoglobin Concentration (MCHC), g/dL, mean (SD)		25	32.1 (1.0)	22	33.4 (1.8)	**0.005** *
Lymphocytes (LYM), x10³/µL, mean (SD)			1.8 (0.9)		8.3 (10.2)	**0.003** *
Platelets (PLT), x10³/µL, median (IQR)			229 (190–266)	23	232 (181–296)	0.563
Mean Corpuscular Volume (MCV), fL, median (IQR)		24	80.7 (76.9–85.5)	22	77.2 (72.3–83.8)	0.176
Mean Platelet Volume (MPV), fL, median (IQR)			8.4 (7.7–9.8)		9.8 (8.5–11.0)	0.054
**Renal Functions**						
Blood Urea Nitrogen, mg/dL, mean (SD)		24	134.3 (37.7)	23	153.7 (32.0)	0.063
Urea, mg/dL, mean (SD)		23	49.5 (24.9)		63.8 (29.8)	0.083
Creatinine, mg/dL, mean (SD)		24	8.8 (2.3)		10.9 (2.6)	**0.005** *
**Electrolytes and minerals**						
Calcium, mg/dL, mean (SD)		24	9.0 (0.9)	23	8.3 (1.2)	**0.033** *
Phosphorus, median (IQR)		22	4.2 (3.7–4.9)	22	5.9 (4.7–8.8)	**0.002** *
Alkaline Phosphatase, U/L, median (IQR)		24	157 (87–266.5)	12	164 (87.5–207)	0.590
Albumin, g/dL, median (IQR)		23	4.0 (3.7–4.0)	23	3.8 (3.7–4.1)	0.587
**Iron studies**						
Serum Iron, µg/dL, median (IQR)		14	47 (36–67)	11	57 (40–72)	0.526
Ferritin, ng/mL, median (IQR)			457 (262–804)	12	153 (46.5–587)	0.066
Total Iron-Binding Capacity (TIBC), µg/dL, mean (SD)			219.2 (47.8)	11	231.8 (43.4)	0.503
C-Reactive Protein (CRP), mg/L, median (IQR)		5	10.2 (5.9–15.8)	2	21.4 (6.8–36)	0.857

* significant p-value.

#### Comparing CCBs and control group

26 patients on HD receiving CCBs were compared to 21 patients not receiving antihypertensive medications (control group). The two groups were comparable in age (51.0 ± 14.4 vs. 49.8 ± 13.1 years, *p* = 0.777) and sex distribution (male 53.8% vs. 47.6%, *p* = 0.772). Hepatitis C infection (3.9% vs. 15.0%, *p* = 0.303), DM (19.2% vs. 9.5%, *p* = 0.436), and cardiac disease (15.4% vs. 0%, *p* = 0.117) were comparable between groups. As expected, hypertension prevalence was markedly higher in the CCBs group (96.2% vs. 38.1%, *p* < 0.001).

Mean dialysis duration was numerically longer among CCBs users compared to the control group (9.4 ± 5.5 vs. 7.8 ± 4.8 years), though this difference did not reach statistical significance (*p* = 0.363). Systolic and diastolic blood pressures were comparable between groups (SBP = 138.6 ± 18.8 vs. 138.1 ± 13.5 mmHg, *p* = 0.942; DBP = 82.6 ± 11.4 vs. 77.9 ± 11.6 mmHg, *p* = 0.243).

Regarding vascular access characteristics, including AVF vintage, laterality, and access type were comparable between the groups (*all p* > 0.05). Additionally, no significant difference was observed in the proportion of patients with a history of failed AVFs (33.3% vs. 40.0%, *p* = 0.674). Complication patterns such as wall thickening, thrombosis, and calcification were comparable (*p* = 0.412) (Supplementary Table 1).

#### Association between CCBs vs BBs and AVF flow rates and complications

Table 3 summarizes the multivariable regression analyses examining the association between antihypertensive medication use and AVF flow rate. In both adjusted models, the AVF flow rate did not differ significantly between CCBs and BBs groups. In Model 1 (adjusted for age, sex, smoking, and dialysis duration), the coefficient was −8.41 mL/min (95% CI: −432.07 − 415.25; *p* = 0.968) and was 147.28 (–298.42 − 592.98; *p* = 0.507) in Model 2 (adjusted for cardiac disease and DM).

**Table 3.** Association of antihypertensive medications with AVF parameters.

Outcome	Model	Antihypertensive vs control		CCBs vs control		CCBs vs BBs	
		Coefficient/OR (95% CI)	P value	Coefficient/OR (95% CI)	P value	Coefficient/OR (95%CI)	P value
AVF Flow Rate (ml/min)	1	−346.56 (−746.04, 52.92)	0.088	−355.18 (−884.35,173.98)	0.179	−8.41(−432.07,415.25)	0.968
	2	−85.88 (−456.18, 284.42)	0.644	−198.15 (−652.08, 255.79)	0.381	147.28 (−298.42, 592.98)	0.507
Any AVF Abnormality*	1	1.41(0.34, 5.86)	0.638	1.21 (0.20, 7.34)	0.835	1.88 (0.37, 9.58)	0.448
	2	0.93 (0.31, 2.78)	0.895	0.83 (0.22, 3.13)	0.778	1.08 (0.31, 3.72)	0.902
Previous Failed AVF*	1	0.55 (0.12, 2.40)	0.423	0.91 (0.17, 4.91)	0.912	0.02 (0.001, 0.34)	**0.007** ^**^
	2	0.56 (0.15, 2.10)	0.390	1.07 (0.24, 4.75)	0.925	0.18 (0.03, 1.06)	0.058
AVF Peak Systolic Velocity (cm/s)	1	−102.51 (−258.68, 53.65)	0.190	−134.61 (−368.46, 99.24)	0.225	−69.04 (−207.73, 69.64)	0.314
	2	−12.46 (−104.19, 79.27)	0.785	−23.32 (−151.47, 104.83)	0.706	−14.56 (−111.17, 82.05)	0.760
Draining Vein PSV (cm/s)	1	−68.01 (−155.23, 19.22)	0.124	−67.96 (−186.88, 50.97)	0.251	−39.85 (−115.33, 35.63)	0.292
	2	−20.51 (−89.44, 48.42)	0.554	−15.63 (−116.00, 84.74)	0.753	−18.64 (−84.05, 46.77)	0.567
Aneurysmal Dilation*	1	2.94 (0.41, 20.96)	0.281	3.66 (0.27, 50.42)	0.332	1.13 (0.22, 5.76)	0.882
	2	1.62 (0.39, 6.67)	0.506	2.33 (0.41, 13.40)	0.341	0.60 (0.13, 2.70)	0.502
Thrombotic Complication*	1	1.42 (0.26, 7.71)	0.688	1.10 (0.16, 7.61)	0.925	2.57 (0.36, 18.55)	0.350
	2	0.50 (0.14, 1.86)	0.303	0.36 (0.06, 2.28)	0.276	1.32 (0.26, 6.78)	0.738

* Odds ratios reported for binary outcomes.

^**^ Significant *p*-value, and denote the single statistically significant *p*-value (*p* = 0.007, CCBs vs. BBs, Previous Failed AVF, Model 1).

Model 1: adjusted for age, sex, smoking, dialysis duration.

Model 2: adjusted for diabetes, cardiac disease, calcium abnormality.

There was no significant difference in the odds of any AVF abnormality or thrombotic complications. However, CCBs users had significantly lower odds of a history of previous failed AVF in Model 1 (OR = 0.02; 95% CI: 0.001–0.34; *p* = 0.007), with this association approaching significance in Model 2 (*p* = 0.058). All other AVF characteristics and secondary outcomes were not significantly different between groups (Table 3).

#### Association between CCBs vs. no antihypertensive therapy and AVF flow rate and complications

Compared with patients who were not on antihypertensive therapy, CCBs users showed no statistically significant differences in primary or secondary outcomes across all models. Although the mean AVF flow rate was numerically lower in CCBs users (Model 1: −355.18 mL/min; *p* = 0.179; Model 2:–198.15 mL/min; *p* = 0.381), these findings did not reach statistical significance. Similarly, the rates of AVF abnormalities, previous access failure, thrombotic events, and aneurysmal dilation were comparable between groups (all *p* > 0.05). Association between CCBs vs. any antihypertensive and AVF flow rate.

Patients on antihypertensive treatment, when compared to controls, had no significant differences in AVF flow rate or secondary flow parameters in either Model 1 (–346.56 mL/min; *p* = 0.088) or Model 2 (–85.88 mL/min; *p* = 0.644).

There were no significant associations between antihypertensive use and odds of any AVF abnormality, previous AVF failure, thrombotic complications, or aneurysmal dilation across all models (*p* > 0.05 for all).

#### Predictors of AVF flow rate

In the univariate linear regression (Table 4), age was inversely associated with AVF flow rate (β = −13.8 mL/min/year, 95% CI −23.8 to −3.7; *p* = 0.008), and this remained significant after multivariable adjustment (β = −14.2 mL/min, 95% CI −25.4 to −3.1; *p* = 0.013).

**Table 4.** Univariate and multivariable predictor models of AVF outcomes.

	Flow rate (ml/min)				Other failed AVF			
Predictors	Univariate Linear Analysis		Multivariable Linear Analysis		Univariate Logistic Analysis		Multivariable Logistic Analysis	
	β (95% CI)	P-value	β (95% CI)	P-value	OR (95% CI)	P-value	OR (95% CI)	P-value
Model 1: Demographic Predictors								
Age (years)	−13.77 (−23.83, −3.71)	**0.008** ^**^	−14.24 (−25.36, −3.13)	**0.013** ^**^	0.95 (0.91, 0.99)	**0.024** ^**^	0.96 (0.91, 1.00)	0.052
Sex (Male vs. Female)	−16.78 (−323.94, 290.37)	0.914	21.80 (−301.27, 344.87)	0.893	0.83 (0.28, 2.47)	0.742	0.47 (0.12, 1.79)	0.269
Smoking (Yes vs. No)	−209.11 (−702.60, 284.38)	0.401	−231.29 (−693.63, 231.05)	0.320	1.41 (0.31, 6.29)	0.656	0.95 (0.18, 5.00)	0.948
Dialysis Duration (mo)	−2.18 (−33.91, 29.54)	0.891	−13.32 (−42.65, 16.00)	0.366	0.94 (0.84, 1.06)	0.300	0.96 (0.85, 1.08)	0.492
Model 2: Comorbid Predictors								
DM	362.48 (−66.74, 791.71)	0.097	305.96 (−132.40, 744.33)	0.168	1.41 (0.31, 6.29)	0.656	1.55 (0.31, 7.77)	0.597
Hypertension	−220.89 (−611.23, 169.45)	0.263	−149.94 (−563.85, 263.97)	0.472	0.30 (0.08, 1.21)	0.090	0.29 (0.07, 1.22)	0.090
Cardiac Disease	−268.79 (−722.13, 184.55)	0.241	−259.56 (−729.82, 210.71)	0.274	0.88 (0.16, 4.80)	0.885	1.30 (0.22, 7.65)	0.775
Calcium abnormalities	79.59 (−236.58, 395.75)	0.617	39.73 (−278.05, 357.51)	0.803	0.67 (0.22, 2.05)	0.485	0.75 (0.23, 2.46)	0.638
Model 3: Vascular Access Predictors								
– 2–5 years vs. < 2 yrs	16.27 (−608.58, 641.12)	0.958	4.36 (−731.14, 739.87)	0.990	2.33 (0.22–25.24)	0.486	3.58 (0.27–47.31)	0.333
– > 5 years vs. < 2 yrs	170.16 (−454.69, 795.00)	0.584	364.42 (−367.71, 1096.55)	0.317	0.88 (0.07–11.31)	0.919	1.55 (0.11–22.61)	0.751
AVF Site (Left vs. Right)	−120.70 (−530.24, 288.84)	0.558	−53.37 (−673.65, 566.92)	0.861	0.70 (0.20–2.44)	0.579	0.90 (0.12–6.79)	0.916
AVF Type (Radiocephalic vs. Brachiocephalic)	−3.57 (−355.53, 348.39)	0.984	−172.88 (−791.46, 445.70)	0.572	0.14 c	0.074	Omitted*	–

* Omitted in the multivariable logistic model because it perfectly predicted failure.

^**^ Significant *p*-value, and denote the three statistically significant *p*-values (*p* = 0.008, *p* = 0.013, and *p* = 0.024, all corresponding to Age).

Neither sex, smoking, dialysis duration, nor comorbidities such as diabetes, hypertension, or cardiac disease showed significant associations with flow rate (all *p* > 0.05).

#### Predictors of a history of previous AVF failure

In logistic regression, increasing age was also linked to lower odds of previous AVF failure (unadjusted OR = 0.95, 95% CI 0.91–0.99; *p* = 0.024; adjusted OR = 0.96, 95% CI 0.91–1.00; *p* = 0.052).

AVF type (radio-cephalic vs. brachiocephalic) showed a non-significant trend toward reduced odds of failure (OR = 0.14, 95% CI 0.02–1.20; *p* = 0.074) but was omitted from the multivariable model due to perfect prediction. No other demographic, comorbid, or vascular variables were independent predictors of AVF failure.

## Discussion

In this cross-sectional study of patients on maintenance HD, we investigated the impact of the vasodilator effect of antihypertensive medication on AVF flow rate, and complications. Our findings suggest that antihypertensive therapy overall is not significantly associated with adverse or beneficial changes in AVF hemodynamics or vascular access complications. However, in a subgroup comparison between classes, CCBs group was independently associated with a significantly lower likelihood of a history of prior AVF failure when compared to BBs use. The lack of significant association between antihypertensive use and AVF flow rate or complications in our study aligns with previous studies that found no clear benefit or harm of specific antihypertensive classes on AVF outcomes. For example, the Hemodialysis Fistula Maturation (HFM) study, which followed 602 participants either receiving maintenance dialysis or expected to start dialysis within three months of planned AVF surgery, found that none of the pre-surgery antihypertensive medication classes, including CCBs and BBs, were significantly associated with changes in AVF diameter or blood flow measured at 6 weeks following surgery. Furthermore, they concluded that the CCBs may be associated with a lower risk of overall AVF maturation failure [11]. Similarly, observational data from a smaller prospective cohort study following 73 HD participants suggested that the administration of antihypertensive medications did not demonstrate a significant correlation with either the AVF maturation outcome or the time required for maturation [20]. While CCBs promote peripheral vasodilation [25,26] and theoretically improve arterial inflow, their role in venous remodeling or AVF structural stability is less clear [27,28].

Interestingly, although systolic blood pressure was significantly lower in CCBs users compared to BBs users, this did not translate into differences in AVF flow or complication rates. This highlights the complexity of blood pressure regulation in dialysis patients and suggests that vascular access outcomes may not be solely dependent on systemic BP control but also on local vascular factors and vessel wall integrity [29].

In our study, no significant associations were found between any antihypertensive use and AVF secondary outcomes such as thrombotic complications, aneurysmal dilation, or AVF PSV. These complications are likely multifactorial, involving local inflammation, repeated cannulation trauma, and systemic factors such as mineral bone disease, which may not be directly influenced by antihypertensive pharmacotherapy [30].

Regarding predictors of AVF outcomes in our study, while DM showed a nonsignificant trend toward higher AVF flow rate (β = 306; *p* = 0.17) we observed a significant inverse association between age and AVF flow rate (−14 mL/min/year). This may reflect age-related vascular changes, such as reduced vessel elasticity and endothelial function, which could compromise fistula maturation. In contrast to Lin et al. (1998) study that assessed the feasibility of creating a radio cephalic hemodialysis fistula in elderly and diabetic patients, prospectively studied 176 patients undergoing the first permanent vascular access creation and followed the outcome of fistula until primary failure or success. They found that old age or diabetes per se did not significantly predispose a new fistula to primary failure, but concurrent old age and diabetes markedly increase the risk, and concluded that, a good primary outcome of newly created radio cephalic fistula and adequate dialysis *via* fistula were demonstrated for elderly and diabetic patients. However, they recommended the need for further study regarding the longevity of fistula in elderly and diabetic patients [31].

Surprisingly, older patients significantly showed 5% lower odds of previous history of other AVF failures in the univariate analysis. However, this did not remain significant in the multivariable model and even trended lower (OR 0.96 per year), suggesting that once established, AVFs in older patients may remain relatively stable.

A primary strength of this study is the comprehensive evaluation of specific antihypertensive classes (CCBs vs. BBs) through standardized Doppler ultrasound protocols. Rather than relying solely on clinical examination or binary patency outcomes (open vs. closed), we utilized quantitative hemodynamic metrics – specifically AVF flow rate and Peak Systolic Velocity (PSV) – in conjunction with clinical complications (e.g., thrombosis and aneurysm). This granular approach allows for a more detailed assessment of how these common medications correlate with both the functional performance and structural integrity of the vascular access in a real-world hemodialysis population providing a holistic evaluation of AVF performance. The use of multivariable regression allowed control for key confounders such as age, comorbidities, and dialysis duration, enhancing the reliability of observed associations. The findings offer practical implications for antihypertensive selection in HD patients, supporting individualized therapy without compromising vascular access function.

Several limitations should be considered. First, the study design is cross-sectional, limiting our ability to infer causality. Second, the sample size was initially powered based on an assumed standard deviation (SD) of 400 mL/min. However, our observed data and other contemporary studies (e.g., the HFM study) demonstrate a larger SD of approximately 600 mL/min. This higher-than-expected variability suggests that our study was likely underpowered to detect a 300 mL/min difference in AVF flow rates between CCBs users and controls. Therefore, the lack of statistical significance in our primary outcome that may result from the study being underpowered rather than a true lack of effect should be interpreted with caution, and larger multi-center trials are required to definitively determine the impact of CCBs on fistula hemodynamics.

Third, the classification of antihypertensive medications was based on current use and did not account for dose, duration, or adherence. Additionally, we did not assess arterial stiffness or perform flow-volume mapping beyond ultrasound-based metrics. Importantly, the nature of the study did not allow for precise tracking of medication initiation relative to the date of AVF surgery; it is possible that some participants commenced CCB therapy after the initial maturation phase. Consequently, our findings reflect a cross-sectional association between CCB use and maintained AVF flow rather than the drug’s direct impact on the physiological process of early post-operative remodeling.

Finally, in the subgroup analysis of CCBs and BBs, we acknowledge a longer duration of dialysis, with lower prevalence of Hepatitis C infection compared to BBs users. Although adjustments were made for dialysis vintage and key clinical variables, residual confounding cannot be excluded, and the potential of selection bias remains.

## Conclusions

Overall, our findings suggest that the use of CCBs does not appear to negatively impact AVF flow rates or clinical maturation in this cohort. While the study was underpowered to recommend a definitive antihypertensive hierarchy, these results provide preliminary evidence that CCBs may be utilized safely without compromising vascular access function. Antihypertensive selection should remain individualized, focusing on cardiovascular stability and blood pressure control until larger prospective trials are conducted to confirm these findings and to guide individualized antihypertensive strategies in patients with arteriovenous access.

## Summary

In summary, antihypertensive medication use was not independently associated with AVF flow rate measured by Color Doppler ultrasound or complications. However, CCBs were associated with significantly reduced odds of a history of previous AVF failure compared to BBs. Older age emerged as the only consistent independent predictor of reduced AVF flow rate and lower risk of a history of previous AVF failure.

## Supplementary Material

Supplemental Material

Supplemental Material

## Data Availability

The data that support the findings of this study are available from the corresponding author, EE, upon reasonable request.

## Data access

All authors had full access to the data and take full responsibility for the integrity of the data and the accuracy of the data analysis.
